# Incidence of pacing‐induced cardiomyopathy in pacemaker‐dependent patients is lower with leadless pacemakers compared to transvenous pacemakers

**DOI:** 10.1111/jce.14814

**Published:** 2020-11-25

**Authors:** Reynaldo Sanchez, Anish Nadkarni, Benjamin Buck, Georges Daoud, Tanner Koppert, Toshimasa Okabe, Mahmoud Houmsse, Raul Weiss, Ralph Augostini, John D. Hummel, Steven Kalbfleisch, Emile G. Daoud, Muhammad R. Afzal

**Affiliations:** ^1^ Division of Cardiovascular Medicine, Davis Heart and Lung Research Institute The Ohio State University Wexner Medical Center Columbus Ohio USA

**Keywords:** atrioventricular node ablation, leadless pacemaker, pacing‐induced cardiomyopathy, right ventricular pacing, transvenous pacemaker

## Abstract

**Introduction:**

Frequent right AQ4ventricular pacing (≥40%) with a transvenous pacemaker (TVP) is associated with the risk of pacing‐induced cardiomyopathy (PICM). Leadless pacemakers (LPs) have distinct physical and mechanical differences from TVP. The risk of PICM with LP is not known. To identify incidence, predictors, and long‐term outcomes of PICM in LP and TVP patients.

**Methods:**

The study comprised all pacemaker‐dependent patients with LP or TVP who had left ventricular ejection fraction (LVEF) of ≥50 from 2014 to 2019. The incidence of PICM (≥10% LVEF drop) was assessed with an echocardiogram. Predictors for PICM were identified using multivariate analysis. Long‐term outcomes after cardiac resynchronization (CRT) were assessed in both groups.

**Results:**

A total of 131 patients with TVP and 67 with LP comprised the study. All patients in the TVP group and the majority in the LP group underwent atrioventricular node ablation. The mean follow‐up duration in TVP and LP groups was 592 ± 549 and 817 ± 600 days, respectively. A total of 18 (13.7%) patients in TVP and 2 (3%) in LP developed PICM after a median duration of 254 (interquartile range: 470) days. The incidence of PICM was significantly higher with TVP compared with LP (*p* = .02). TVP as pacing modality was a positive (odds ratio [OR]: 1.07) while age was negative (OR: 0.94) predictor for PICM on multivariable analysis. Both patients in LP and all except two in the TVP group responded to CRT.

**Conclusion:**

Incidence of PICM is significantly lower with LP compared with TVP in pacemaker‐dependent patients. Age and TVP as pacing modality were predictors for PICM.

## 1 INTRODUCTION

Leadless pacemakers (LPs) offer an excellent alternative to transvenous pacemakers (TVPs) in patients needing single ventricular pacing and avoid the complications inherent to the transvenous devices including pocket infections, lead failures, chronic venous occlusions, tricuspid valve regurgitation, pocket hematomas, and pneumothoraces.^1^, ^2^ The Micra transcatheter catheter pacing system (Micra‐TPS; Medtronic Inc.) is the only commercially available LP. Before the recent approval of atrioventricular (AV) synchronous Micra (Micra AV), only 15% of the patients were eligible for LP. Based on the existing LP use trend, most of the patients implanted with LP are either those who need infrequent ventricular pacing or those who have a normal ventricular function and undergo AV node ablation for atrial fibrillation with a rapid ventricular response.

Patients needing frequent right ventricle (RV) pacing have a 10%–15% incidence of pacing‐induced cardiomyopathy (PICM).^3^, ^4^ Certain clinical predictors for PICM have been identified and include wide QRS duration at baseline, RV pacing burden, and reduced ventricular function before pacemaker implantation. Some studies have suggested a role of tricuspid regurgitation (TR) and apical location of pacing lead in the RV as contributory to the development of PICM; however, the data are discordant.^5^, ^6^ Incidence of PICM has not been reported for LP. There are several physical and mechanical differences between TVP and LP. LP is preferentially implanted in the septum and may be immune to lead‐related development of TR. Implications of these factors for the development of PICM in patients with LP are not known.

This study aimed to compare the incidence and predictors of PICM in patients with LP and TVP.

## 2 METHODS

### 2.1 Patient population

The study comprised all patients with a normal left ventricular ejection fraction (LVEF ≥ 50%) at baseline who underwent LP or TVP implantation from February 2014 to June 2019. The data were obtained by a retrospective review of prospectively maintained databases for implantable devices at Ohio State University. The Institutional Review Board of the Ohio State University approved the study. Patients with a history of cardiac resynchronization therapy (CRT) and recovered LVEF who underwent extraction followed by single ventricle pacing were excluded from the study. In addition, patients who were not 100% pacemaker dependent during follow‐up were excluded.

### 2.2 Indications for pacemaker implantation

Guideline‐directed indications for pacemaker implantation were followed.^7^ The choice of LP versus TVP was operator discretion. At author's institution, the use of LP has steadily increased over time for single ventricular pacing. LP is particularly preferred in elderly patients and those with multiple comorbidities, chronic kidney disease, and dialysis. The majority of the patients in the TVP group had dual‐chamber devices and underwent AV node ablation for rate control in the setting of permanent AF.

### 2.3 Implantation procedure

Implantation of LP and TVP, as well as AV node ablation, was performed according to the standard technique described previously.^8^, ^9^ AV node ablation was performed either concurrently or during follow‐up. Briefly, all patients underwent an ipsilateral venogram for the TV group before the axillary or subclavian vein access. The site of RV lead implantation was based on the discretion of the operator. For the LP group, a femoral approach was used for venous access. Deployment location was assessed in both right anterior oblique and left anterior oblique views. A chest X‐ray was performed 2–4 h after the procedure in both groups to assess for pneumothorax (TV group) and lead/LP location. For patients undergoing concurrent AV node ablation, the devices were programmed for a lower rate of 80 beats per minute in a VVIR mode. All patients were seen in the device clinic after 2–4 weeks to assess pectoral incision, femoral access, and pacing parameters. For patients with AV node ablation, the lower rate was programmed to 60 or 70 based on operator preference.

### 2.4 Data collection

Data were collated by retrospective chart review. Data included baseline demographics, clinical characteristics, echocardiographic parameters at baseline and follow‐up after pacemaker implantation, electrocardiographic features at baseline and after implantation of a pacemaker, location of RV lead or LP and acute and long‐term procedure‐related complications. A censor date corresponding to the most recent device interrogation, last known clinical follow‐up was recorded for all patients who did not develop PICM during the study period. For assessment of QRS duration at baseline and with pacing, a 12‐lead electrocardiogram was used. For determination of implant location, a chest X‐ray and cine image at end of the procedure were used.

### 2.5 Primary outcome

Incidence of PICM defined as a 10% decrease in LVEF during follow‐up in both groups was assessed as the primary outcome. Patients with alternative explantation of cardiomyopathy, such as the development of sarcoidosis, myocarditis, myocardial infarction, were excluded from the primary outcome analysis.

### 2.6 Clinical management and follow‐up for patients with PICM

Management of patients developing PICM was done according to institutional protocol and physician preference. Where indicated, the implantation of CRT was pursued according to the standard techniques.^10^

### 2.7 Statistical analysis

Categorical data are represented by numbers and percentages. Continuous data are represented as means and *SD*s. Student's *t*‐test was used to compare continuous variables and *χ* ^2^ test was used to compare categorical variables with *α* = .05 for these tests. Predictors of PICM were assessed in a stepwise manner: univariate analysis was first performed to identify candidate predictors; candidate predictors with *p* < .20 were promoted into a multivariate analysis and were incorporated into the final significance if *p* < .05.

## 3 RESULTS

### 3.1 Study population

A total of 131 patients with TVP and 67 with LP fulfilled the inclusion criteria. Baseline demographics, clinical characteristics, and relevant echocardiographic parameters for patients in both groups are given in Table 1. These characteristics were somewhat similar with some differences. Notably, fewer female patients received LP compared with TVP. The prevalence of CAD was higher in the TVP group.

**Table 1:** Baseline characteristics of TVP and LP groups

Variables	TVP (*n* = 131)	LP (*n* = 67)	*p* Value
Age	74 ± 10	73 ± 16	.59
Race (Caucasian)	123 (94)	60 (90)	.27
Gender, female	96 (73)	31 (46)	.0003
Body mass index (kg/m^2^)	29 ± 9	30 ± 7	.42
History of CAD	70 (53)	18 (27)	.0001
History of hypertension	110 (84)	52 (77)	.33
History of CHF	49 (37)	24 (36)	.87
History of diabetes	37 (28)	27 (40)	.35
Dialysis	7 (5)	3 (4)	1
CKD (GFR < 30)	27 (21)	18 (27)	.37
LVEF within 6 m before pacemaker implantation	59 ± 5	57 ± 5	.01
TR within 6 m before pacemaker			
None or trace	17 (13)	5 (7)	.34
Mild	42 (32)	11 (16)	.02
Moderate	29 (22)	23 (34)	.08
Severe	27 (21)	12 (18)	.7
Unknown	16 (12)	16 (24)	.04

*Note*: Data are given in mean ± *SD* and *n* (%).

Abbreviations: CAD, coronary artery disease; CHF, congestive heart failure; CKD, chronic kidney disease; GFR, glomerular filtration rate; LP, leadless pacemaker; TR, tricuspid regurgitation; TVP, transvenous pacemaker.

### 3.2 Implant and pacing characteristics

All patients in the LP and TVP groups were successfully implanted with pacemakers without any acute device‐related complication. Indication of pacemaker implantation, duration of QRS with and without pacing, and implant location are given in Table 2. Mainly, permanent AF with rapid ventricular response was the most common indication of pacemaker implantation in both groups. The majority of the patients in both groups had concurrent AV node ablation. Patients in the LP group who did not have AV node ablation were also completely pacemaker dependent. The duration of QRS at baseline before pacemaker implantation was significantly longer in the LP group than TVP (115 ± 37 vs. 96 ± 22; *p *= *.0001*). Similarly, the duration of QRS after pacing was also significantly longer with LP (164 ± 24 vs. 155 ± 25; *p* = .01). The majority of the LP were implanted in the mid‐septal (58%) or apical septal (31%) location. The majority of the TVP was implanted in the apex (67%) followed by the apical septum (18%; Table 2).

**Table 2:** Implant and pacing characteristics of TVP and LP groups

Variables	TVP (*n* = 131)	LP (*n* = 67)	*p* Value
Implant indications			
AF with RVR	131	47 (70)	.01
AF with slow ventricular rate	0	12 (18)	.01
Sinus rhythm with high grade AV block	0	8 (12)	.1
Native QRS duration	96 ± 22	115 ± 37	.0001
Paced QRS duration	155 ± 25	164 ± 24	.01
Implant location in right ventricle			
Apex	88 (67)	7 (10)	.0001
Apical septum	23 (18)	21 (31)	.03
Mid septum	12 (9)	39 (58)	.0001
Basal septum/RVOT	0		
Anterior wall	3 (2)		
Unknown	5 (4)		

*Note*: Data are given in mean ± *SD* and *n* (%).

Abbreviations: AF, atrial fibrillation; LP, leadless pacemaker; RVOT, right ventricular outflow tract; RVR, rapid ventricular response; TVP, transvenous pacemaker.

### 3.3 Primary outcome

Mean follow‐up duration after AV node ablation in TVP and LP groups was 592 ± 549 and 817 ± 600 days, respectively. Overall, 18 (13.7%) patients in TVP and 2 (3%) in LP developed PICM after a median duration of 254 (interquartile range [IQR]: 470) days after implantation. The two patients in the LP group developed PICM after 180 and 350 days. The median duration before PICM in TVP group was 194 (IQR: 429). The incidence of PICM was significantly higher with TVP compared with LP (*p* = .02).

### 3.4 Acute and long‐term procedure‐related complications

Incidence of acute periprocedural and follow‐up complications (>30 days) was similar in both groups as outlined in Table 3. Notably, one patient in the LP group had pericardial effusion noted immediately after deployment. A total of 45 (67%) patients in LP and 86 (67%) in the TVP group had an interpretable echocardiogram within 6 months of pacemaker implantation. At least one degree of TR worsening was seen in 34% in LP and 44% patients with TVP (*p* = .22; Table 3).

**Table 3:** Acute and chronic device‐related complications of TV and LP groups

Variables	TVP (*n* = 131)	LP (*n* = 67)	*p* Value
Acute (0–30 days)			
Pocket hematoma	1 (1.5)	0	1
Groin hematoma	1 (1.5)	2 (3)	1
Device revision	2 (3)	0	1
Device infection	1 (1.5)	0	1
Pericardial effusion	0	1	1
Chronic (>30 days to longest available follow‐up)	2	0	1
Device revision	2	0	1
Device infection Overall	9 (6.4)	3 (4.4)	.75
Worsening of at least one degree of TR within 6 months	58 (44)	23 (34)	.22

*Note*: Data are given as *n* (%).

Abbreviations: LP, leadless pacemaker; TR, tricuspid regurgitation; TVP, transvenous pacemaker.

### 3.5 Multivariable analysis for predictors of PICM

Univariate analysis revealed that age, LVEF at baseline, TVP as pacing modality, and nonseptal location were predictors of PICM. TVP as pacing modality was a positive (odds ratio [OR]: 1.07) while age (OR: 0.94) was a negative predictor for PICM on the multivariable analysis (Table 4).

**Table 4:** Multivariable analysis to assess predictors of PICM in both groups

	Univariate analysis			Multivariate analysis		
Variable	OR	95% CI	*p* Value	OR	95% CI	*p* Value
Age	1.05	1.0–1.80	***.01***	0.94	0.91–0.98	***.01***
CAD	1.05	0.41–2.66	.92			
CHF	0.54	0.19–1.55	.25			
DM	1.76	0.63–4.96	.28			
Female gender	0.65	0.26–1.66	.37			
LVEF at baseline	1.22	1.07–1.39	***.01***			
TVP vs. LP	5.18	1.16–23.03	***.03***	1.07	1.50–48.74	***.02***
QRS at baseline	1.00	0.98–1.02	.96			
QRS with pacing	1.00	0.98–1.02	.68			
Nonseptal location	3.11	1.07–9.02	***.04***			

Abbreviations: CAD, coronary artery disease; CHF, congestive heart failure; CI, confidence interval; CKD, chronic kidney disease; DM, diabetes mellitus; LP, leadless pacemaker; LVEF, left ventricular ejection fraction; OR, odds ratio; PICM, pacing‐induced cardiomyopathy; TR, tricuspid regurgitation; TVP, transvenous pacemaker.

### 3.6 Impact of CRT on PICM in both groups

In patients diagnosed with PICM, the mean LVEF before the upgrade was 35 ± 8. All patients with PICM in both groups underwent an upgrade to a CRT after a median duration of 29 (IQR: 40) days after diagnosis. A repeat echocardiogram performed after a median duration of 147 (IQR: 259) days after upgrade revealed a mean ejection fraction of 48 ± 10. Except for two patients in the TVP group, all patients in both groups had significant improvement (≥10%) in LVEF with the upgrade (*p* = .01). Response to CRT was similar in both LP and TVP groups.

## 4 DISCUSSION

### 4.1 Major findings

The study's major finding is that the incidence of PICM is lower with LP compared with TVP in pacemaker‐dependent patients. Age at pacer implant and TVP as pacing modality was a predictor for PICM in this cohort. Institution of CRT resulted in the normalization of LVEF in the majority of the patients.

### 4.2 PICM in RV pacing

PICM in patients with single ventricle pacing is a well‐known phenomenon. The incidence in various published studies is variable.^4^, ^11^ Prior studies have used variable rate of ventricular pacing as inclusion criteria. Overall, 40% right ventricular pacing is considered a risk factor for the development of PICM.^3^, ^4^ Although not studied systematically, it is conceivable that the development of PICM has a linear relationship with ventricular pacing. Prior studies using a cutoff of 40% used a cumulative burden of ventricular pacing identified on periodic device interrogations. Using a cutoff such as 40% has limitations. It is common to observe that lower programming rates are often set at 60 beats per minute in patients with single ventricle pacemakers. Most of these patients require ventricular pacing at night or during periods of rest when the native heart rate is lower than the minimum programmed rate. The influence of ventricular pacing during various phases of physical activity is not evident. The rationale for including patients with 100% ventricular pacing in this study was to minimize the influence of variable duration, frequency, and timing of single ventricle pacing. This study demonstrated that the development of PICM was significantly lower in patients with LP compared with TVP. According to our knowledge, this aspect of LP has not been reported in the pivotal trial and subsequent studies.^12^, ^13^, ^14^

### 4.3 Predictors of PICM and relevance to LP

#### 4.3.1 Baseline ventricular function

Previous studies have identified various clinical characteristics as predictors of PICM. Baseline ventricular function before implantation of pacemaker has been identified as one risk factor for the development of PICM. This finding is the reason for the current recommendation of implantation of CRT in patients with ventricular dysfunction who are expected to require at least 40% pacing after implantation of a pacemaker.^3^, ^4^ This study aimed to identify the differences in LP and TVP for the development of PICM and thus excluded patients with ventricular dysfunction. The findings of this study clearly demonstrate that the incidence of PICM in patients with LP was significantly lower compared with TVP when baseline ventricular function is normal.

#### 4.3.2 Age

Age was a negative independent predictor for PICM on a multivariable analysis in this study. A small number of PICM patients in the LP cohort did not allow separate assessment of predictors in the LP cohort. These data may suggest that TVP and LP may be comparable for the development of PICM in elderly patients.

#### 4.3.3 Location of LP or pacing lead

This study clearly showed the differences in implantation location for LP and TVP where the majority of the LP were implanted in mid‐septal location. Univariate analysis showed that nonseptal location was a predictor of PICM; however, the multivariable analysis did not show any association. A small number of patients with PICM in the LP cohort is probably the reason for the nonsignificant association in multivariate analysis. It is conceivable that a larger study with more patients with PICM in the LP cohort may delineate the real impact of implant location for the development of PICM. Multiple previous studies have looked at the impact of apical versus septal pacing for the development of PICM.^5^, ^11^, ^15^ Two older studies found an association of apical pacing and PICM.^11^, ^15^ A recent study did not find any association of lead location on the incidence of PICM; however, this study had a relatively shorter follow‐up of 14 months compared with a previous study of similar size with discordant results.^5^

#### 4.3.4 Duration of native and paced QRS

Some previous studies have suggested that the duration of native QRS and paced QRS may impact the development of PICM.^5^ Due to preferential implantation of LP in the septal location, it was conceivable that the duration of paced QRS in patients with LP may be shorter than TVP as seen in previous studies.^16^ This study did not show any significant reduction of QRS duration with septal pacing with LP. A recent study also suggested that interventricular dyssynchrony (aortopulmonary ejection delay: >40 ms) was a predictor of PICM in patients with TVP.^5^ It is likely that interventricular dyssynchrony is less pronounced with septal pacing; however, this study did look at this aspect. It is hypothesis generating and could be assessed in the future investigation in patients with LP.

#### 4.3.5 Role of TR in the development of PICM

Previous studies have shown a harmful effect of TR in patients with otherwise normal ventricular function.^17^ Incidence of newly developed or worsening of at least one degree of TR after single ventricular pacing ranges is as high as 40%.^18^ Role of newly developed or worsening TR as a predictor for PICM has not been examined in the previous studies; however, a significant progression in TR was seen with transvenous leads.^6^ This study showed that the incidence of newly developed or worsening TR was numerically higher with TVP compared with LP (44% vs. 34%); however, it did not achieve statistical significance (*p* = .22). It is conceivable that a larger cohort may be able to delineate the impact of TR in the development of PICM in LP and TVP patients requiring frequent RV pacing.

#### 4.3.6 Management issues for PICM in LP and TVP groups

CRT's role in patients with PICM is very well demonstrated in previous studies.^19^ Majority of the patients who underwent CRT after PICM in this study noted an improvement in LVEF. The only two patients in the LP group who underwent CRT also showed significant improvement in LVEF. In both cases, the LP was abandoned, and a new RV and CS lead was implanted. Although abandoning an LP is an acceptable strategy, retrieval can also be attempted.^9^

#### 4.3.7 Future perspectives

Before the availability of Micra AV, the use of LP was limited to patients who need single ventricle pacing, predominantly patients with infrequent need of pacing and those with permanent AF. Due to recent studies demonstrating the safety of LP over TVP due to the lack of lead and pocket‐related complications, and availability of AV synchronous pacing, it is expected that the use of LP will increase over time. The findings of this study complement the previous studies and highlight another avenue where LP has fared better than TVP.

#### 4.3.8 Limitations

This study has some limitations. (i) The study is a retrospective single‐center study from a high‐volume center and the findings may not be evident in low‐volume centers. (ii) The study is nonrandomized and device selection was based on operator discretion. Despite all these limitations, this is the first study suggesting the superiority of LP compared with TVP for the incidence of PICM and can serve as hypothesis‐generating for larger future studies.

## 5 CONCLUSIONS

This is the first study showing that the incidence of PICM is significantly lower for LP compared with TVP in pacemaker patients with normal ventricular function. Further prospective randomized trial is needed to validate these findings.

## Data Availability

The data that support the findings of this study are available from the corresponding author upon reasonable request.
